# Inhibition of pressure-activated cancer cell adhesion by FAK-derived peptides

**DOI:** 10.18632/oncotarget.20556

**Published:** 2017-08-24

**Authors:** Bixi Zeng, Dinesh Devadoss, Shouye Wang, Emilie E. Vomhof-DeKrey, Leslie A. Kuhn, Marc D. Basson

**Affiliations:** ^1^ Department of Surgery, University of North Dakota School of Medicine and Health Sciences, Grand Forks, North Dakota, United States; ^2^ Department of Biomedical Sciences, University of North Dakota School of Medicine and Health Sciences, Grand Forks, North Dakota, United States; ^3^ Department of Biochemistry & Molecular Biology, Michigan State University, East Lansing, Michigan, United States; ^4^ Department of Computer Science & Engineering, Michigan State University, East Lansing, Michigan, United States

**Keywords:** wound recurrence, FAK, Akt1, mechanotransduction, metastasis

## Abstract

Forces within the surgical milieu or circulation activate cancer cell adhesion and potentiate metastasis through signaling requiring FAK-Akt1 interaction. Impeding FAK-Akt1 interaction might inhibit perioperative tumor dissemination, facilitating curative cancer surgery without global FAK or AKT inhibitor toxicity. Serial truncation and structurally designed mutants of FAK identified a seven amino acid, short helical structure within FAK that effectively competes with Akt1-FAK interaction. Adenoviral overexpression of this FAK-derived peptide inhibited pressure-induced FAK phosphorylation and AKT-FAK coimmunoprecipitation in human SW620 colon cancer cells briefly exposed to 15mmHg increased pressure, consistent with laparoscopic or post-surgical pressures. Adenoviral FAK-derived peptide expression prevented pressure-activation of SW620 adhesion not only to collagen-I-coated plates but also to murine surgical wounds. A scrambled peptide did not. Finally, we modeled operative shedding of tumor cells before irrigation and closure by transient cancer cell adhesion to murine surgical wounds before irrigation and closure. Thirty minute preincubation of SW620 cells at 15mmHg increased pressure impaired subsequent tumor free survival in mice exposed to cells expressing the scrambled peptide. The FAK-derived sequence prevented this. These results suggest that blocking FAK-Akt1 interaction may prevent perioperative tumor dissemination and that analogs or mimics of this 7 amino acid FAK-derived peptide could impair metastasis.

## INTRODUCTION

Disseminated tumor cells are pivotal in cancer metastasis. A one centimeter tumor sheds one million cells into the circulation daily [1]. Surgery may facilitate metastasis. Viable tumor cells [2] can frequently be recovered from the peritoneum [3] or portal or systemic circulation during colon cancer resections [4, 5]. Although it is difficult to distinguish undetected preoperative metastases from metastases arising from tumor cells dislodged by surgical manipulation, 0.2-0.5% of potentially curative surgical resections are marred by surgical wound recurrence [6]. Many more patients develop peritoneal dissemination or circulatory metastasis. Since most cancer patients die of metastasis, not primary tumors, inhibition of metastasis is highly desirable. Perioperative tumor dissemination can turn curative cancer resections into a metastatic fatalities.

Exposing suspended colon cancer cells to 15 mmHg increased pressure promotes cell adhesion to collagen [7, 8], endothelial monolayers, and murine surgical wounds [9, 10]. Pressure also activates adhesiveness in breast cancer [11], squamous cell carcinoma [12] and sarcoma cells [13]. Activating this pathway potentiates peritoneal dissemination directly impacting survival in animal models [9, 10]. Shear similarly activates cancer cell adhesion [8, 14].

Disseminated tumor cells encounter such forces in various milieus. Circulatory pressures range from 5-10 mmHg in the portal vein to 90-120 mmHg in systemic arteries. (Pressures are referenced as gauge pressure, the excess beyond the 760 mmHg atmospheric pressure.) Tumors growing against constrictive stroma exhibit average 15-38 mmHg interstitial fluid pressures [15]. Surgical tumor manipulation generates pressures of 1500 mmHg [16] while irrigation causes shear. Laparoscopic insufflation elevates intra-abdominal pressure by 15 mmHg throughout surgery. Mechanical stimuli, such as pressure [17–19], shear [14, 20], and strain [21, 22], influence physiological and pathological biology.

Pressure and shear activate dual mechanosensitive signaling pathways involving the cytoskeleton and paxillin or Src and phosphatidylinositol 3-kinase (PI3K) respectively [23, 24]. These converge at FAK and Akt1, wherein Akt1 phosphorylates FAK at serines-517/601/695 facilitating FAK activation [25]. This increases β1-integrin affinity and avidity, lowers the ligand threshold required for integrin-mediated cell adhesion, and facilitates tumor dissemination [8]. This signal pathway occurs in suspended cells. Adherent cancer cells, with a different cytoskeletal configuration, respond to increased pressure via a different pathway that triggers proliferation [26]. Because Akt binding to FAK is uncommon in described signal pathways, we sought to prevent FAK activation by targeting this interaction, sparing conventional FAK-activating stimuli, and to inhibit this pathway in a more specific manner than typical FAK inhibitors.

The FAK molecule is divided into the N-terminal erythrocyte band four.1-ezrin-radixin-moesin (FERM) (residues 35-362), the kinase (residues 416-676), and the C-terminal focal adhesion targeting (FAT) (residues 677-1025) domains [27]. The FERM domain provides an interface between membrane and cytoskeletal components and directs FAK activity [27]. The conformation of the kinase domain exhibits little change in transition between active and inactive states and is therefore thought to be controlled through active site occlusion [28]. The FAT domain targets FAK to focal adhesions and is required for integrin-mediated FAK signaling [29]. We have previously shown that FAK-Akt1 binding requires neither the FAK kinase nor FAT domains; the highest Akt1 affinity depends on FERM domain residues 1-126 [30–32]. Using structural analysis and truncations of this fragment of human FAK as our starting point, we dissected the FAK-Akt1 interaction to define a short FAK-derived peptide that interrupts said interaction and demonstrated that this peptide inhibits cancer cell adhesion in vitro and in vivo and improves survival in a murine model of wound recurrence. 15 mmHg increased extracellular pressure was the prototypical force stimulus and wound adhesion the prototypical model for perioperative cancer cell metastasis.

## RESULTS

### GST-FAK fusion proteins pull-down purified and endogenous Akt1, is modulated by mutations, and persists in NT1-2-2 truncations

**Figure 1:**
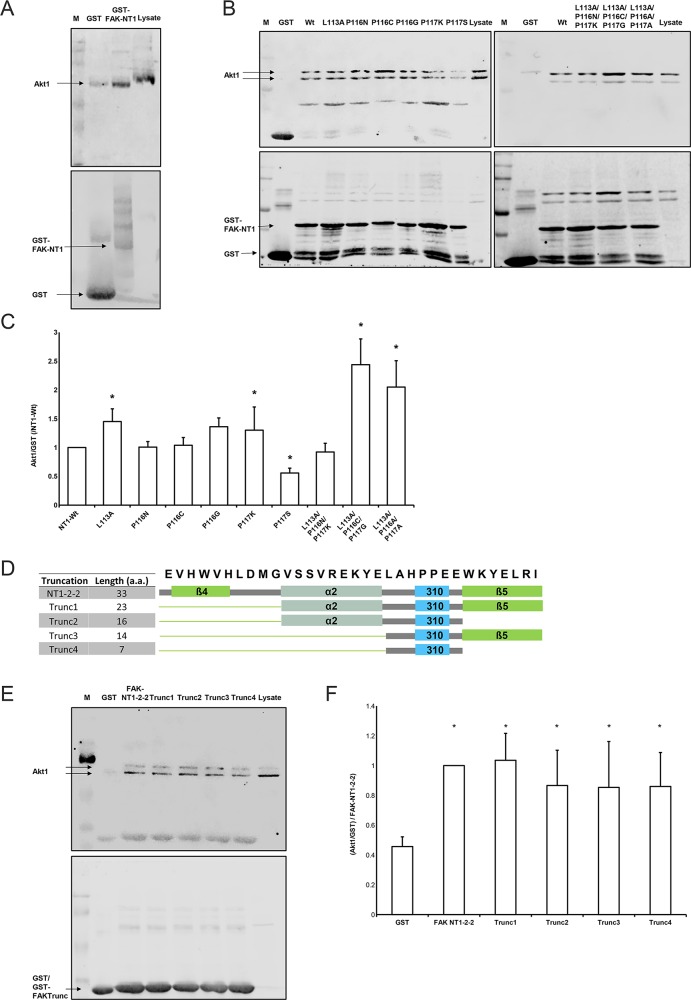
Interaction of Akt1 with FAK truncations **(A)** GST-FAK-NT1 conjugated beads pulled down purified Akt1 (N=3). The western blot shows the amount of Akt1 (prey, 60 kDa) signal relative to the amount of GST-FAK-NT1 (bait, estimated 35 kDa) signal. **(B)** The western blot shows the amount of Akt1 (prey) signal relative to the amount of GST-FAK-NT1 or FAK mutant (bait) signal. **(C)** Densitometric data was analyzed as the percentage of Akt1 signal over GST fusion protein signal, which was then normalized to the wild-type NT1. (n=8-19, ^*^ p<0.05 vs. the GST-FAK NT1 wild-type). **(D)** The amino acid sequence of the NT1-2-2 peptide with the corresponding secondary structures are shown; the β-helices are shown in green, the α-helix in gray, and the short helix in cyan. The cartoon representations of the secondary structures present in each truncation correspond to the constructs found in the table to the left. **(E)** The western blot shows the amount of Akt1 (prey) signal relative to the amount of GST/GST-FAK truncation (bait) signal. The low molecular weight of the truncations impedes the differentiation between GST-FAK truncations and unbound GST tags. **(F)** Densitometric data was analyzed as the percentage of Akt1 signal over GST fusion protein signal and then normalized to the Akt1 pulldown from the NT1-2-2 construct. All truncations pulled down significantly more Akt1 than did the GST control (n = 6, ^*^ p < 0.05 vs. GST). Western blots were probed for Akt1 (top) and GST (bottom). A marker (M) and the amount of Akt1 signal produced by 40 μg of SW620 whole cell lysate control were used as a references.

Based on Western blots comparing band intensity of 0.001-0.05 micrograms/lane of purified Akt1 against the band intensity of 40 or 80 micrograms/lane of cell lysate (not shown), we used 0.3 micrograms of purified Akt1 in pull-down assays to approximate the Akt1 in 1,500 μg of whole cell lysate, the lysate from 1×10^7^ SW620 cells previously used in similar pull-down assays. GST-FAK-NT1-conjugated Sepharose beads pulled down Akt1 after overnight incubation with either cell lysate or purified Akt1 (Figure 1A), suggesting that FAK and Akt1 bind directly without intermediary or scaffolding proteins.

We examined the role of a short helical secondary structure (LAHPPEE) using mutated variants of FAK-NT1 (Figure 1B). FAK-NT1 is a larger truncation of FAK encompassing the NT1-2-2 region of interest; it is sufficient to pull down Akt1 and was chosen as a conservative platform that could support the native folding of the NT1-2-2 region in our mutation assays [31]. Of the nine mutants studied, Akt1 binding affinity was significantly different from that of the wild-type F1 lobe for FAK(L113A), (P116G), (P117S), (L113A/P116C/P117G), and (L113A/P116A/P117A) (Figure 1B, 1C). FAK(P117S) was designed to increase short helix rigidity and lowered Akt1 pull-down (p < 0.05, N=11). Conversely, mutants FAK(L113A), (P116G), (L113A/P116C/P117G), and (L113A/P116A/P117A) aimed to destabilize the region and consequently increased Akt1 pull-down (Figure 1C). Single mutants with altered short helix hydrophobicity, FAK(P116N), (P116C), (P116G), and (P117K), did not change Akt1 pull-down except for FAK(P116G). This FAK(P116G) proline to glycine substitution intended to decrease hydrophobicity but also lowered the propensity for maintaining the wild-type helical structure by Chou-Fasman analysis. Altogether, FAK NT1 pull-down of Akt1 is altered by point mutations to this short helical region.

**Figure 2:**
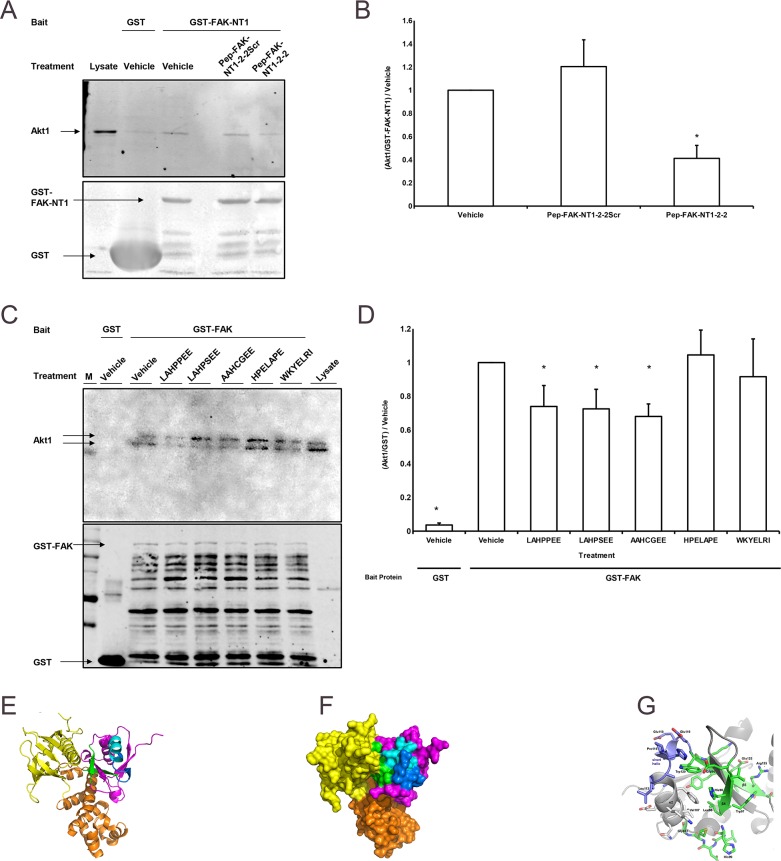
FAK-derived peptides interfere with FAK-Akt1 interaction **(A)** The western blot shows the amount of Akt1 (60kDa) signal relative to the amount of GST (25 kDa) /GST-FAK-NT1 (estimated 35 kDa) signal with a marker (M) and the amount of Akt1 signal produced by 40 μg of SW620 whole cell lysate control as a references. Treatments indicated refer to the interfering peptide or vehicle (water) used. Western blots from the Akt1 pulldown assays were probed for Akt1 (top) and GST (bottom). **(B)** Treatment with the wild-type 33 amino acid peptide (Pep-FAK-NT1-2-2) reduced the amount of Akt1 pulled down by GST-FAK-NT1 vs. vehicle; the scrambled peptide (Pep-FAK-NT1-2-2Scr) did not have any effect (n = 4, ^*^ p < 0.05 vs. vehicle-treated GST-FAK-NT1). **(C)** Studies parallel to those in Figure 4B, but which used GST-FAK as bait and interfering peptides 7 amino acids in length, showed decreased Akt1 pulldown with the addition of wild type or mutant FAK peptides. The GST probed western (bottom) shows more bands as the full-length GST-FAK (150 kDa) yields more break down products. **(D)** Incubation with the wild-type (LAHPPEE) or mutant (LAHPSEE and AAHCGEE) peptides reduced Akt1 pulldown compared to the vehicle control. No such effect was seen after incubation with the scrambled (HPELAPE) or the β-strand (WKYELRI) control (n = 12-14, ^*^ p < 0.05 vs. vehicle treated GST-FAK). All peptides were used at a concentration of 160 μM. Ribbon **(E)** and surface **(F)** depictions of the crystal structure of the FAK FERM domain containing the F1 (magenta, green and blue), F2 (orange), and F3 (yellow) lobes (from PDB entry 2AL6 [36]), rendered by PyMOL (v. 1.8.2.2; Schrodinger LLC, NY). **(G)** Close-up of the FAK NT1-2-2 peptide region based on the crystal structure of chicken FAK, which is highly similar in sequence. Relative to (E) and (F), the view in panel (G) is rotated by 180° about the z-axis (perpendicular to the plane of the page), to better view the LAHPPEE epitope (residues 113-117). All renderings of the NT1-2-2 segment show secondary-structures β4 and β5 in green, α2 in pale blue, and the short PPE helix in dark blue.

To investigate the importance of individual subdomain structures within NT1-2-2 in FAK-Akt1 binding, we generated four variants that successively excluded secondary structures in the N- and C-terminal of the short helical segment while preserving the short helix itself (Figure 1D). We challenged these truncated versions of NT1-2-2 by Akt1 pull-down. We observed some non-specific GST-binding of Akt1, but NT1-2-2 pulled down much more. Truncation did not interfere with the Akt1-binding of the larger sequence (Figure 1E). The appearance of Akt1 as a doublet here and elsewhere (34-37) is considered by the manufacturers of the Akt1 antibody to be the product of high levels of phosphorylation on Akt1 (personal communication, Julie Diamond, Cell Signaling Technology). Western blot comparisons of Akt1 from cell lysate (doublet) with purified Akt1 (single band) show the heavier band of the Akt1 doublet as matching the band produced by the purified Akt1. The densitometry in this figure and in Figure 2 show data from the heavier, top band that corresponds to the band seen with purified Akt1. However, separate densitometric analysis of the lower band in each case yields similar, statistically significant results (data not shown).

### NT1-2-2 derived peptides inhibit FAK pull-down of Akt1

To minimize the effect of non-specific binding to GST, we next used the larger FAK-NT1 (relative to GST) as bait and assessed the effects of the NT1-2-2 FAK truncations as interfering peptides. The wild-type 33 amino acid peptide (Pep-FAK-NT1-2-2) reduced binding between GST-FAK-NT1 and Akt1. A scrambled 33 amino acid control peptide containing the same amino acids in a different order. (Pep-FAK-NT1-2-2Scr) did not (Figure 2A, 2B). We subsequently used full length GST-FAK as bait to further validate the ability of the interfering peptide to block Akt1 interaction with the entire FAK molecule. Because the seven amino acid sequence from the short helix (LAHPPEE) seemed sufficient for Akt1 binding in Figure 1E above, we focused on this seven amino acid sequence and mutants thereof. Bacterial production of GST and GST-FAK does not occur at the same rate despite being under the control of identical promoters. In Figure 2C, lane 2 used GST alone as the bait protein and produced a large, low weight band while samples that used GST-FAK as bait (lanes 3-8 show multiple bands, which may be the breakdown products of the original GST-FAK construct. We matched the GST in all lanes to provide a conservative negative control. Despite the relatively higher amount of GST bait protein over GST-FAK, all GST-FAK constructs pulled down significantly more Akt1 than the GST alone. Wild-type LAHPPEE and mutant (LAHPSEE and AAHCGEE) versions of the FAK peptide reduced Akt1 pulldown by human wild type GST-FAK in the presence of vehicle alone (Figure 2C, 2D). Neither a scrambled version of the short helix (HPELAPE) nor a peptide derived from the β-strand secondary structure C-terminal adjacent to the short helix (WKYELRI) interfered with pulldown. The location of the LAHPPEE peptide within the FAK molecule is shown in ribbon (Figure 2E) and surface (Figure 2F) renderings of the crystal structure of the FAK FERM domain (PDB entry 2AL6 (30)). The NT1-2-2 segment is located mostly on the surface of the F1 domain (magenta, green, and blue) and is colored as shown in Figure 1D, with β4 and β5 in green, α2 in pale blue, and the short PPE helix in dark blue. A magnified version of the FAK NT1-2-2 peptide region (Figure 2G) shows the β4 and β5 in green, α2 in gray (left), and the LAHPPEE short helix in dark blue (upper left).

### Both pressure-induced phosphorylation of FAK, but not Akt1 or GSK-3β, and pressure-induced HA-FAK, Akt1 coimmunoprecipitation are inhibited in SW620 infected with adenovirus expressing FAK-derived peptides

**Figure 3:**
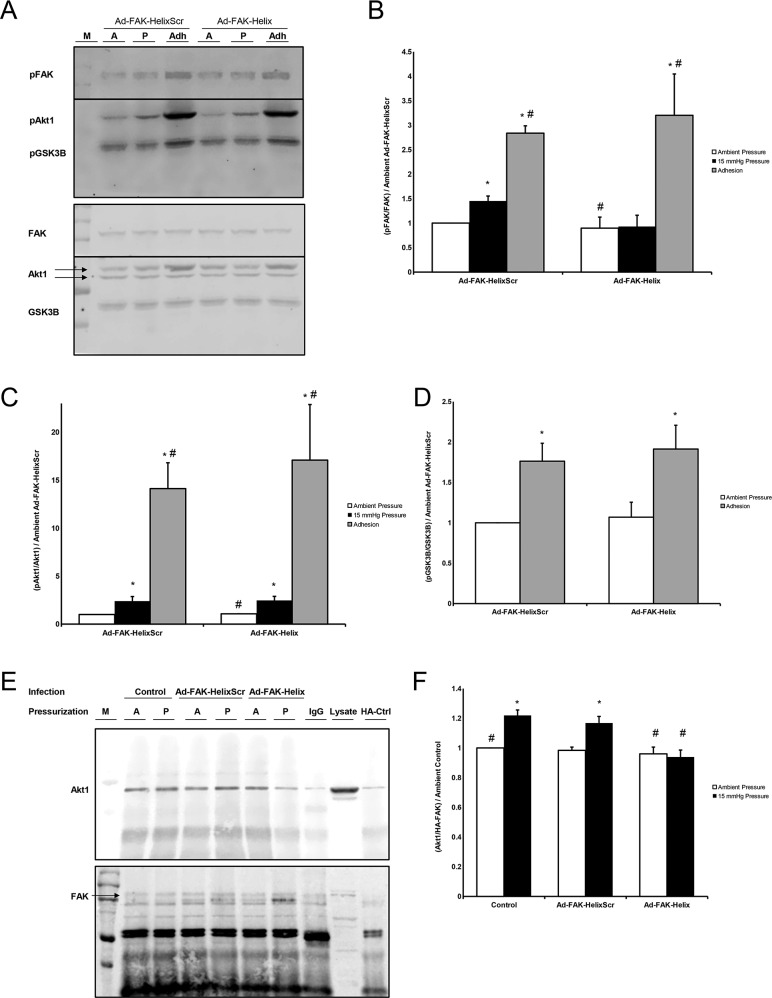
**(A)** Lysate from Ad-FAK-HelixScr and Ad-FAK-Helix infected cells exposed to ambient (A) or 15 mmHg (P) while in suspension, or allowed to adhere to collagen I (Adh) were probed for phospho- (top) and total (bottom) FAK, Akt1, and GSK3B. Blots were cut at the level of 75 kD,, and the higher weight bands were incubated with pFAK/FAK (125 kDa) antibodies while the lower weight bands received pAkt1/Akt1 (60 kDa) and pGSK3B/GSK3B (46 kDa) probes. **(B)** In suspended cells, pFAK increased following 15 mmHG exposure vs. exposure to ambient atmosphere in Ad-FAK-HelixScr, but not Ad-FAK-Helix infected cells. In contrast, adhesion increased FAK phosphorylation over suspended cells at ambient pressure in both Ad-FAK-HelixScr and Ad-FAK-Helix infected cells. **(C)** Both Ad-FAK-HelixScr and Ad-FAK-Helix virus infected cells exhibited increased Akt1 phosphorylation after exposure to 15 mmHg pressure as well as after adhesion. **(D)** GSK-3β phosphorylation also increased in both the virus treated cells in response to adhesion; however, in pressure treated groups, GSK-3β phosphorylation decreased in the Ad-FAK-HelixScr but not the Ad-FAK-Helix virus treated cells (n = 4-8, ^*^ p < 0.05 vs. ambient Ad-FAK-HelixScr, ^#^ p < 0.05 vs. 15 mmHg Ad-FAK-HelixScr). **(E)** HA/HA-FAK coimmunoprecipitated Akt1 (top) to produce a western signal which was normalized to the respective amount of FAK signal (bottom). The samples are grouped by viral infection, uninfected (Control), Ad-FAK-HelixScr, or Ad-FAK-Helix, and then subdivided by exposure to ambient (A) or 15 mmHg pressure (P). All cells were transfected with HA-FAK except the HA-Ctrl cells which were transfected with a plasmid expressing the HA tag alone. A marker (M) and 40 μg of SW620 whole cell lysate were used as a references for Akt1 and FAK. **(F)** Exposure to pressure increased Akt1 coimmunoprecipitation in control SW620 cells or cells infected with Ad-FAK-HelixScr. Pressure did not increase Akt1 coimmunoprecipitation in SW620 cells infected with Ad-FAK-Helix (n = 6, ^*^ p < 0.05 vs. ambient pressure, ^#^ p < 0.05 vs. 15 mmHg Ad-FAK-HelixScr).

We created adenoviral vectors to deliver the wild type sequence LAHPPEE (Ad-FAK-Helix) or the scrambled HPELAPE (Ad-FAK-HelixScr) into intact human SW620 colon cancer cells and assessed pressure-induced signaling. 15mmHg increased pressure stimulated FAK Tyr397 phosphorylation (145±10%) in cells infected with the scrambled Ad-FAK-HelixScr control vs. cells at ambient pressure (Figure 3A, 3B). In contrast, pressure did not stimulate FAK Tyr397 phosphorylation in cells infected with Ad-FAK-Helix, overexpressing the native FAK-derived seven amino acid sequence. q-RT-PCR analysis of the helical and scrambled peptide messages suggested similar expressions in infected cells (not shown). Pressure-induced FAK and Akt1 phosphorylation is initiated by cytoskeletal mechanosensing in suspended cells [8] independent of traditional adhesion-induced signaling, which begins with surface integrin binding and progresses inward activating associated proteins. Adhesion to collagen I induced FAK Tyr397 phosphorylation in both Ad-FAK-HelixScr and Ad-FAK-Helix infected cells similarly (Figure 3B), suggesting the specificity of the effect of LAHPPEE in inhibiting FAK-Akt1 interaction.

Because pressure stimulates Akt1 Ser473 phosphorylation and Akt1 activation before Akt1 phosphorylates FAK [25], we predicted that LAHPPEE would not interfere with other aspects of Akt1 signaling. Neither virus inhibited pressure-induced Akt1 Ser473 phosphorylation (consistent with the model that Akt1 activation by pressure occurs upstream of Akt1-FAK interaction) or adhesion-induced Akt1 Ser473 phosphorylation (Figure 3C). We examined phosphorylation of the Akt target protein GSK-3β [33] after adhesion to further evaluate the potential for peptide overexpression to modulate Akt1 downstream signaling. Cell adhesion stimulated GSK-3β Ser9 phosphorylation similarly in Ad-FAK-HelixScr-infected and Ad-FAK-Helix infected cells (Figure 3D). Interestingly, pressure had an unanticipated inhibitory effect on GSK-3β phosphorylation.

We have previously demonstrated that pressure stimulates FAK-Akt1 interaction in intact SW620 cells, transfected with HA-FAK before immunoprecipitation with anti-HA to amplify the signal produced by basal FAK-Akt1 interaction [8, 25]. We now performed parallel studies to validate our in vitro findings in intact cells, using adenoviral infection to introduce FAK-derived peptides (Figure 3E). Consistent with published observations [25], pressure increased co-precipitating Akt1 from uninfected SW620 cells vs. cells at ambient atmospheric pressure. Infection with Ad-FAK-Helix blocked this effect. Ad-FAK-HelixScr did not (Figure 3F). Due to the size of the FAK-derived peptide as well as the region of FAK from which it was derived, Western blots using FAK antibody probes are unable to detect the presence of our peptide. However, q-RT-PCR analysis of the helical and scrambled peptide messages suggested similar expression in infected cells (not shown).

### FAK-derived peptide overexpression prevents pressure-induced SW620 cell adhesion but does not affect proliferation

**Figure 4:**
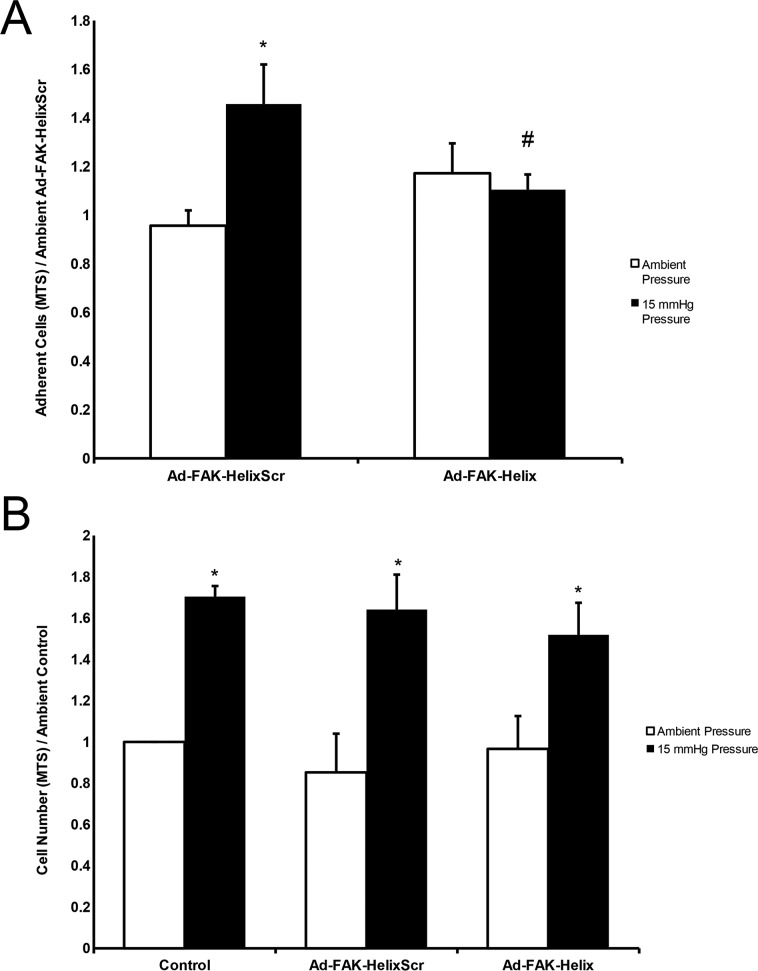
A FAK-derived peptide blocks pressure stimulation of adhesion but not pressure stimulation of proliferation **(A)** Exposure to elevated pressure increased adhesion by SW620 cells infected with the Ad-FAK-HelixScr virus vs. ambient pressure. Adhesion by cells infected with the Ad-FAK-Helix virus did not change in response to increased pressure and was not different at ambient pressure from the adhesion of cells infected with Ad-FAK-HelixScr at ambient pressure. (n = 8, ^*^ p < 0.05 vs. the paired ambient pressure group, ^#^ p < 0.05 vs. 15 mmHg Ad-FAK-HelixScr). **(B)** In adherent cells, exposure to increased pressure stimulated cell proliferation in control (uninfected), Ad-FAK-HelixScr infected, and Ad-FAK-Helix infected SW620 cells (n = 4, ^*^ p < 0.05 vs. the paired ambient pressure group).

We hypothesized that peptide overexpression would similarly inhibit pressure-stimulated adhesion, the downstream consequence of pressure-activated FAK phosphorylation. Equal numbers of virus-treated cells were seeded onto collagen-I-coated plates under ambient or increased pressure for 30 minutes. The plates were washed in blinded fashion to remove nonadherent cells. The remaining adherent cells were quantified by MTS assay. Pressure-induced adhesion was inhibited by Ad-FAK-Helix infection but not by Ad-FAK-HelixScr (Figure 4A).

Increased pressure stimulates cancer cell proliferation by a different mechanism [10, 19, 26]. We examined the effect of Ad-FAK-Helix on ambient and pressure-stimulated SW620 proliferation to determine whether observed changes in FAK signaling and adhesion might reflect non-specific disruption of cell physiology. Neither proliferation at ambient pressure nor the mitogenic effect of increased pressure was affected by either Ad-FAK-HelixScr or Ad-FAK-Helix. (Figure 4B).

### Infection with adenovirus expressing FAK-derived peptides inhibits pressure-stimulated wound-implantation

**Figure 5:**
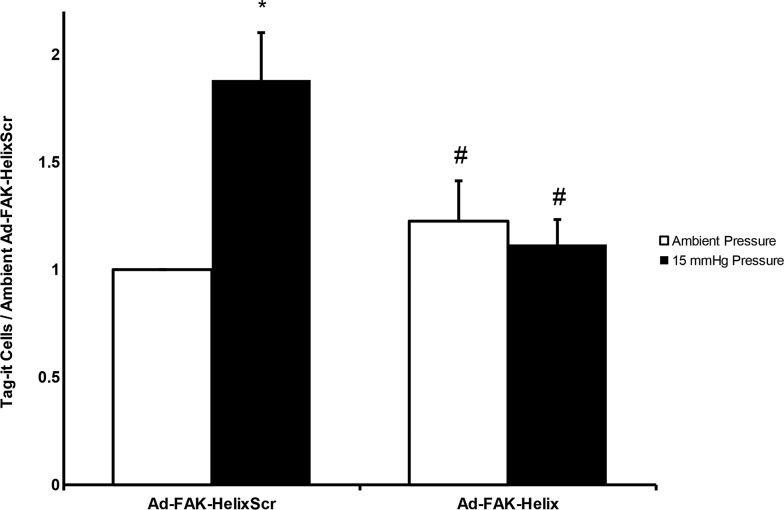
A FAK-derived peptide blocks pressure stimulation of adhesion of cancer cells to murine surgical wounds Tag-it-labeled, Ad-FAK-HelixScr virus treated cells displayed increased wound implantation under elevated pressure conditions, after assay by complete excision of the wound and flow cytometric quantitation of labelled cells in wound tissues. Treatment with the Ad-FAK-Helix blocked this effect (n = 14, ^*^ p < 0.05 vs. the paired ambient pressure group, ^#^ p < 0.05 vs. 15 mmHg Ad-FAK-HelixScr).

Since physiologic tissues are more complex than purified matrix proteins, we investigated SW620 cell adhesion to surgical wounds in BALB/c mice. SW620 cells infected with either Ad-FAK-HelixScr or Ad-FAK-Helix were labeled with Tag-it Violet dye, exposed to ambient or 15 mmHg increased pressure for 30 minutes in suspension and seeded into standardized murine surgical wounds. After 30 minutes, copious irrigation removed non-adherent cells as in surgical settings. After sacrifice and wound excision, adherent SW620 cells were distinguished from mouse tissue by fluorescence-activated cell sorting (FACS) for the Tag-it Violet dye. The dye was non-toxic and did not alter proliferation (not shown). Pressure activation increased Ad-FAK-HelixScr-infected cell implantation into wound tissue. Ad-FAK-Helix infection blocked this effect (Figure 5).

### Infection with adenovirus expressing FAK-derived peptide reduces subsequent murine tumor development by pre-exposure of implanted tumor cells to elevated pressure

**Figure 6:**
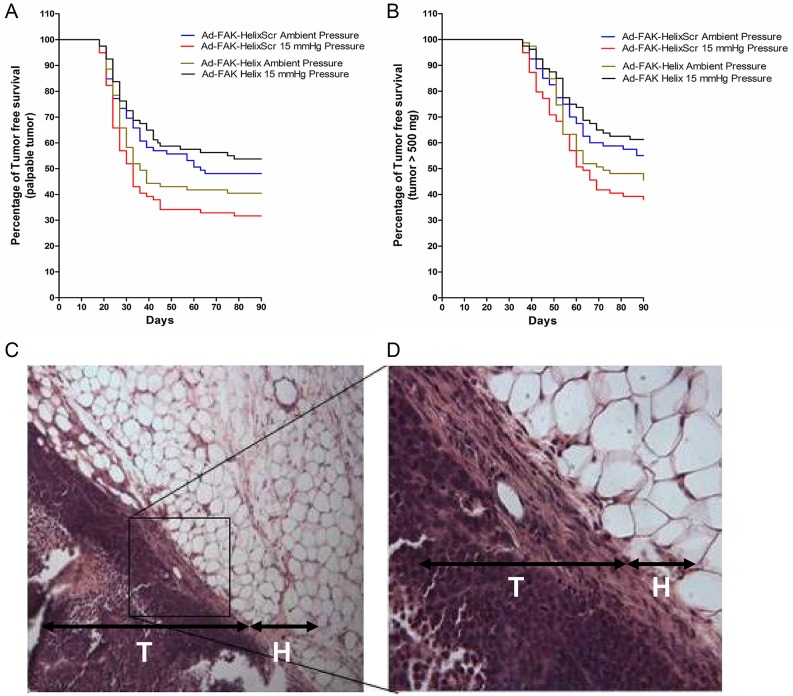
Effects of transient expression of a FAK-derived peptide on subsequent tumor development in a model of surgical wound occurrence Murine tumor development and tumor-free survival were evaluated after exposing standardized surgical wounds to SW620 tumor cell suspensions for 30 minutes and then irrigating copiously before wound closure. Before implantation, the cells were infected with Ad-FAK-HelixScr or Ad-FAK-Helix and exposed to ambient pressure or 15 mmHg increased pressure. **(A, B)** The Kaplan-Meier graphs document palpable tumor development and population survival (as represented by the absence of tumors 500 mg in mass) over time. A significant decrease was seen between the ambient and pressure groups in the average tumor-free survival time (ambient, 60.2 days (CI [53.5, 66.9]); pressure, 48.3 days (CI [41.6, 54.9]) (hazard ratio, 1.57 (CI [1.05, 2.35])) and average survival time with < 500 mg tumor-burden (ambient, 74.0 days (CI [69.5, 78.4]); pressure, 66.5 days (CI [61.9, 71.1]) (hazard ratio, 1.64 (CI [1.07, 2.50])) in mice implanted with Ad-FAK-HelixScr cells (p < 0.05, N=79-81). Mice that received Ad-FAK-Helix cells showed no decreases in mean average tumor-free survival between ambient (55.0 days (CI [48.2, 61.8]) and pressure (64.2 days (CI [57.6, 70.7]) groups (hazard ratio, 0.69 (CI [0.45, 1.07])), and average time with < 500 mg tumor burden increased similarly (ambient, 71.4 days (CI [67.1, 75.7]); pressure, 76.6 days (CI [72.5, 80.7]) (hazard ratio, 0.65 (CI [0.41, 1.03])). Hazard ratios calculate the effect of pressure exposure relative to paired ambient controls. ^*^ indicates p < 0.025 vs paired ambient pressure control. **(C, D)** Tissue sections from the murine wound sites implanted with SW620 colorectal adenocarcinoma cells. H, hypodermis; T, tumor cell layer. Original magnification, 100X (C). Original magnification, 400X (D).

We next investigated whether such differences in cell adhesion alter tumor development. Using similar methodology, suspended cells from each of four conditions (ambient or increased pressure, infected with Ad-FAK-HelixScr or Ad-FAK-Helix) were relabeled to blind the surgical investigator and seeded into surgical wounds in mice. After 30 minutes, the wounds were washed six times with warm PBS and closed. The mice were observed for 90 days during which tumors were assessed as palpable or non-palpable (Figure 6A) and palpable tumors were measured to provide objective data (Figure 6B). Mice were euthanized at a 500 mg tumor burden per veterinary recommendations. In the mice implanted with Ad-FAK-HelixScr cells, 52% from the ambient pressure group eventually developed palpable tumors, with an average tumor-free survival time of 60.2 days, and a mean survival time with < 500 mg tumor-burden of 74.0 days. In the mice that received pressure-activated Ad-FAK-HelixScr cells, 68% developed palpable tumors. Average tumor-free survival and survival with < 500 mg tumor burden decreased to 48.3 and 66.5 days, respectively. Log-rank analysis of both the time to palpable tumor and survival with < 500 mg tumor burden curves demonstrated statistically significant effects of pressure pre-activation in mice that received Ad-FAK-HelixScr cells (p < 0.05, N=79-81). In contrast, pressure pre-activation did not worsen survival in mice that received Ad-FAK-Helix cells. The mean average tumor-free survival was 55.0 days and 64.2 days for mice receiving Ad-FAK-Helix cells previously exposed to ambient or increased pressure, respectively. Similarly, the survival time with < 500 mg tumor burden increased from 71.4 days in the ambient pressure group to 76.6 days in the increased pressure group and the percentage of palpable tumors decreased from 58% in the ambient group to 46% in the increased pressure group (these changes were not statistically significant). With both the time till palpable tumor and survival with < 500 mg tumor burden, group comparisons (Holm-Sidak method) between the ambient and pressure arms of the model showed only significant differences in the Ad-FAK-HelixScr cells (p < 0.025, N=79-81). Within the Ad-FAK-HelixScr cells, the increased hazard in the pressure-treated group when compared to the group exposed to ambient pressure was 1.57 for the time till palpable tumor and 1.64 for the time of survival with < 500 mg tumor-burden. When these comparisons were made with the Ad-FAK-Helix cells, the hazard in the pressure-treated group decreased to 0.69 for the time till palpable tumor and to 0.65 for the time of survival with < 500 mg tumor-burden. Histological examination of the growths confirmed the presence of tumors both through the contrast against the normal epithelial and fascial tissue (Figure 6C) and through the structure of the tumor itself (Figure 6D).

## DISCUSSION

Physical forces evoke signaling responses across diverse cells by different mechanisms. The potentiation of adhesion in suspended cancer cells by a force-activated pathway represents a target for inhibiting metastasis, and the uncommon FAK-Akt1 interaction essential for this pathway seems an attractive target because blocking it may not affect other FAK signaling. Our results demonstrate that Akt1 interacts with FAK directly without an intermediary protein, likely via a short helix on the surface of the FAK F1 lobe, and that this FAK-Akt1 interaction can be blocked by peptides derived from said F1 lobe. Indeed, adenoviral delivery of this peptide into intact cancer cells blocks both pressure-activated signaling and consequent increases in cell adhesion without interfering with other aspects of FAK or Akt1 signaling. Finally, while adenoviral peptide delivery may not be a practical therapeutic modality, our findings suggest that interventions using or mimicking the FAK-derived peptide may translate to in vivo models and increase tumor-free survival by mitigating pressure-stimulated tumor adhesion.

FAK-Akt1 signaling has recently been described in several contexts with respect to malignancy. FAK [34] and Akt1 [35] are important kinases in a common pathway in cancer cells, and increased extracellular pressure induces Akt1 to phosphorylate FAK at serines 517/601/695 and threonine 600 [25]. The GST-FAK pull-down of Akt1 from cell lysate and of purified Akt1, as well as co-precipitation of FAK and Akt1 from intact cells suggests a direct interaction between FAK and Akt1 independent of scaffolding proteins. Considered with our previous data characterizing FAK as a substrate of Akt1, this pull-down data provides the impetus behind our efforts to identify the region of FAK responsible for binding Akt1 [25]. Successive truncations revealed a seven residue sequence (residues 113-119 LAHPPEE) containing a short helix on the surface of the F1 lobe of the FAK FERM domain that was capable of pulling down Akt1. Furthermore, mutations in this region proved sufficient to alter Akt1 affinity. To address the size discrepancy between the GST tag and our shorter truncations, we reproduced the pull-down studies between wild-type GST-FAK and Akt1 with the addition of peptides modeled after the FAK short helix. The ability of this region to inhibit GST-FAK pull-down of Akt1 as an interfering peptide is consistent with its ability to bind Akt1 as a bait protein. The specificity of this interference is suggested by the inability to produce such effects using either a scrambled version of this short helix or a FAK-derived β-strand, the secondary structure found COOH-terminally adjacent to the short helix in the wild-type FAK sequence.

Pressure requires FAK-Akt1 interaction to stimulate FAK tyrosine-397 autophosphorylation and activation [25]. The FAK F1 lobe appears sufficient to bind Akt1 [31]. The FERM domain includes three lobes: F1 (residues 35-130), F2 (residues 131-255), and F3 (residues 256-362) (Figure 2E, 2F). The F2 lobe binds the kinase domain to fold the entire FERM domain over the kinase, inhibiting FAK catalytic activity by active site denial [28]. The F3 lobe contains a site homologous to other FERM domains that, upon activation, binds cytoplasmic tails of β-integrins and ICAM-2 [36]. It is interesting that the F1 lobe mediates FAK-Akt1 interaction, not the better characterized F2 and F3 lobes, because the F2 lobe regulates FAK activation *following* cell adhesion [36], while pressure activates FAK in suspended cells *before* adhesion [37]. Similarly, the potential protein interaction site of the F3 lobe is occluded in inactivated FAK, precluding it from participating in pressure-induced FAK-Akt1 interactions that cause FAK autophosphorylation [8]. Changes to F1 lobe residues that do not physically contact tyrosine-397 can activate FAK [36]. Mutation of lysine-38, which is topographically distant from tyrosine-397 in crystal structures, may promote tyrosine-397 phosphorylation by destabilizing the FERM-linker interaction [38]. These data support a model in which the FAK-Akt1 interaction alters the F1 lobe to autophosphorylate tyrosine-397.

Consistent with the effects of pharmacologic Akt blockade [8, 25], adenoviral delivery of the short helix peptide blocked pressure-induced FAK tyrosine-397 phosphorylation and FAK-Akt1 coimmunoprecipitation. However, this short helix peptide interrupted FAK-Akt1 interaction while preserving Akt1 kinase activity and downstream GSK phosphorylation. Together with the decrease in pressure-induced FAK-Akt1 association and FAK activation, these observations suggest that the short helix peptide interferes with Akt1 binding to FAK, not Akt1's catalytic competence. We noted incidentally that pressure itself reduced levels of pGSK-3β Ser9 compared to the ambient pressure control. The cause and significance of this is outside the scope of the present study. However, metastatic dissemination is increasingly viewed through a lens of epithelial to mesenchymal and mesenchymal to epithelial transitions (EMT/MET), which has been reported to involve GSK-3β. Active GSK-3β phosphorylates the transcription factor Snail, marking Snail for degradation, and allowing the cell to express epithelial traits. Phosphorylated GSK-3β is inactive and unable to cause the degradation of Snail so that Snail is then free to drive the expression of the mesenchymal traits that allow the cell to migrate, invade, and survive anoikis [39, 40]. Thus, the pressure-stimulated decrease in pGSK-3β that we observed may then correspond to an increase in GSK-3β activity and the subsequent expression of epithelial markers such as E-cadherin which forms cell-cell adhesions [41]. The ability of the short helix peptide to block the pressure-induced inhibition of pGSK-3β could be attributed to the available pool of Akt1 generated by decreased FAK-Akt1 interactions. While the expression of mesenchymal traits aids tumor cells in the process of dissemination, the reexpression of epithelial markers may facilitate the subsequent attachment of disseminated tumor cells to surrounding organ parenchyma as they form secondary tumors. Further studies may examine how increased extracellular pressure interacts with this process.

As for the molecular data, the inhibition of pressure-induced cell adhesion by the short helix peptide was consistent with previous work using less specific pharmacologic agents [8, 9, 25]. Little difference was seen between the basal levels of ambient pressure cell adhesion to collagen I or murine surgical wounds in the groups that received the wild-type short helix peptide and those that received its scrambled control, but Ad-FAK-Helix prevented the stimulation of adhesion by pressure. Indeed, in the tumor progression model, treatment with the virally delivered peptide not only prevented the reduction of tumor-free survival by pressure but increased tumor-free survival in the mice implanted with Ad-FAK-Helix-infected SW620 cells activated with increased pressure compared to those preincubated only at ambient pressure. We previously observed a similar trend when blocking the pressure pathway with a high dose of colchicine [9]. This possible reversal raises the possibilities of a minor counterregulatory pathway that awaits exploration.

Viral toxicity seems unlikely to explain our findings. Control cells were similarly infected, and Ad-FAK-Helix-infected adherent SW620 cells continued basal proliferation and responded to increased pressure with increased proliferation, similar to uninfected cancer cells [19, 26, 42]. This not only suggests the Ad-FAK-Helix-infected cells’ continued viability but also their ability to respond to other mechanotransduced pathways. Because adenoviral infection was transient and proliferation unaffected, neither host effects nor long-term effects on the tumor cells seem likely to contribute to the effects of peptide delivery on tumor development that were constrained to the initial adhesive event.

To power our study, we sought a tumor prevalence of 50% in the ambient control group, which came at the expense of replicating the low baseline incidence of clinically significant tumor recurrence in surgical wounds. However, physical forces activate tumor cell adhesiveness not only in the context of wound implantation but also peritoneal implantation [43] and distant metastasis [44], which are much more common. This in vivo study explored pressure-induced adhesion in the context of wound recurrence following surgical resections, but raise the question of whether manipulation of FAK-AKt1 interaction might also influence metastasis to other sites. For instance, we have previously reported that this pathway potentiates metastatic adhesion in a model of peritoneal wound recurrence (9). This awaits further study beyond the scope of the current investigation. In addition, the magnitude of pressure effects on FAK phosphorylation and adhesion are admittedly small. However, others have previously studied signaling events and differences in adhesion of similar magnitude in other contexts [45–47] More importantly, these relatively small changes in signaling or adhesion translate to potentially clinically relevant differences in percent tumor free survival that could substantially exceed the incremental benefit of some new antineoplastic cytotoxic agents.

These results suggest that perturbing FAK-Akt1 interaction, by mimicking the structure of a small segment of the F1 FAK lobe, can abate the sensitivity of suspended malignant cells to mechanical signals, potentially mitigating both the biochemical and the clinical consequences of this force-activated pathway. Such a resultant decrease in FAK and Akt1 activation and inhibition of cell adhesion could attenuate the metastatic potential of shed tumor cells during surgery and increase tumor-free survival. Potential toxicity and off-target effects limit the clinical utility of other methods to inhibit this force-activated adhesion pathway, such as high dose colchicine, disruption of the cytoarchitecture, or non-specific FAK and Akt inhibitors. Preventing FAK-Akt1 interaction without interfering with other aspects of FAK or Akt1 signaling might eventually achieve the desired effect with less compromise of other cell function and less toxicity.

## MATERIALS AND METHODS

### Structure-based design of peptidyl epitopes to compete with FAK for binding Akt1

The crystal structure of human FAK (PDB 2AL6) and preliminary data showing Akt1 pulldown by a truncated 33 amino acid segment of the F1 lobe of FAK designated NT1-2-2 [30, 32] suggested that the NT1-2-2 region of FAK binds Akt1 through a short helical secondary structure accessible from the protein surface. The 33-residue peptide, NT1-2-2 (residues 94-126, EVHWVH....WKYELRI) includes the second and fourth strands from a small β sheet in FAK (labelled β4 and β5 in Figure 1D, based on their order in the sequence of PDB 2AL6 [36]. This peptide does not include the third strand, which is needed for β-sheet integrity, so NT1-2-2 cannot mimic an intact β-sheet. However, the NT1-2-2 peptide does immunoprecipitate Akt1 (Figure 1A). This suggests that the structurally self-determinate helical region in the NT1-2-2 peptide is the epitope involved in Akt1 binding, formed by the α2 helix plus a single turn of helix formed by residues 116-118 (PPE). Because hydrophobic interactions are important in protein-protein interfaces, we designed peptide variants centered on the hydrophobic C-terminal end of α2, followed by the PPE motif: LAHPPEE (residues 113-117). Consideration of statistical amino acid preferences to occur in α helices, β sheets and reverse (β) turns was augmented by Sequery and SSA analysis [48, 49] of the preferred 3D conformations of tetrapeptide sequences in this region (e.g., LAHP, AHPP, HPPE, etc.) across a representative set of 4300 non-homologous structures in the Protein Data Bank. We designed mutants of FAK (shown as the LAHPPEE sequence for simplicity) or free peptides for competition with FAK as follows:

- L113A: AAHPPEE - Enhanced α helical preference
- P116N: LAHNPEE - Similar α helical, β turn preference in the PPE region, increased polarity
- P116C: LAHCPEE - Structurally labile, greater hydrophobicity
- P116G: LAHGPEE - Stronger turn preference, greater flexibility, less hydrophobicity
- P117K: LAHPKEE - Structurally labile, enhanced polarity
- P117S: LAHPSEE - More structurally labile and polar
- Triple mutant L113A, P116N, P117K: AAHNKEE - Enhanced helicity and polarity
- Triple mutant L113A, P116C, P117G: AAHCGEE - More structurally labile and hydrophobic
- Triple mutant: L113A, P116A, P117A: AAHAAEE - More helical and hydrophobic

### Generation and expression of GST fusion proteins

Bacterial expression vector pGEX-4T1 (GE Healthcare, Munich, Germany) was used as a template to generate mutated and truncated human FAK as GST (Glutathione S-transferase) fusion proteins. Point mutations (L113A, P116C, P116G, P116N, P117K, and P116S) and triple mutants (L113A/P116N/P117K, L113A/P116C/P117G, L113A/P116A/P117A) were generated using the Quick Change II XL Site-Directed Mutagenesis kit (Agilent Technologies, (Santa Clara, CA). Truncations were generated through PCR using forward and reverse primers to direct truncation (Table 1). PCR products were introduced into the pGEX-4T1 template between 5′ EcoRI and 3′XhoI sites. Plasmids were purified via MiniPrep (QIAGEN, Valencia, CA) before sequencing. BL21 competent E. coli (New England Biolabs, Ipswich, MA) were transformed with appropriate plasmids, and IPTG-induced.

**Table 1:** PCR forward primers for FAK truncations

Truncation name	Secondary-structure(s) truncated	Forward primer (*Eco*RI restriction site underlined)	Reverse primer (*Xho*I restriction site underlined)
Truncation 1	β-strand 4	5′-CCGGAATTCGTCTCCAGTGTGAGGGAGAAGTATGAGCTTGCTCACCCACCA-3′	5′-CCGCTCGAGAATTCTCAATTCATATTTCCACTCCTCTGGTGGGTGAGCAAG-3′
Truncation 2	β-strand 4,β-strand 5	5′-CCGGAATTCGTCTCCAGTGTGAGGGAGAAGTATGAGCTTGCT-3′	5′-CCGCTCGAGCTCCTCTGGTGGGTGAGCAAGCTCATACTTCTC-3′
Truncation 3	β-strand 4,α-helix 2	5′-CCGGAATTCCTTGCTCACCCACCAGAGGAGTGGAAATAT-3′	5′-CCGCTCGAGAATTCTCAATTCATATTTCCACTCCTCGGT-3′
Truncation 4	β-strand 4,β-strand 5,α-helix 2	5′-CCGGAATTCCTTGCTCACCCACCAGAGGAG-3′	5′-CCGCTCGAGCTCCTCTGGTGGGTGAGCAAG-3′

### Glutathione S-transferase pull-down

Glutathione-Sepharose-4B beads (30 μl) (GE Healthcare Life Science, Pittsburgh, PA) were conjugated with GST (expressed protein from a 250 μl bacterial pellet per 30 μl of beads) or recombinant GST-tagged (expressed protein from a 3 ml bacterial pellet per 30 μl of beads) [25] and incubated with lysate from 2×10^7^ Caco-2 or SW620 cells (1500 μg protein) or purified Akt1 (0.35 μg) (Origene, Rockville, MD) overnight at 4°C. Similar incubation for two hours at 4°C was performed with FAK-derived peptides (95% purity by HPLC) (Peptide 2.0, Chantilly, VA) reconstituted in sterile water and mixed with conjugated beads and cell lysate for a final concentration of 160mM before overnight Akt1 incubation. Bound protein was eluted for western analysis [25].

### Adenovirus vector construction and production

cDNA coding a seven amino acid segment from the F1 lobe of FAK (FAK-Helix, a.a. 113-119 LAHPPEE) and a scrambled version of this sequence (FAK-HelixScr, HPELAPE) were cloned in-frame into the MCS region of separate pShuttle-CMV vectors (Agilent, Santa Clara, CA) using forward primers that added 5′-NotI and reverse primers that added 3′-HindIII restriction sites (FAK-Helix forward, 5′-CCGTCGACGCGGCCGCATGCTTGCTCACCCACCAGAGGAGTAA-3′ | FAK-Helix reverse, 5′-TCTTATCTAGAAGCTTTTACTCCTCTGGTGGGTGAGCAAGCAT-3′) (FAK-HelixScr forward, 5′-CCGTCGACGCGGCCGCATGCACCCAGAGCTTGCTCCAGAGTAA-3′ | FAK-HelixScr reverse, 5′-TCTTATCTAGAAGCTTTTACTCTGGAGCAAGCTCTGGGTGCAT-3′). The PCR did not use template DNA as forward and reverse primers collectively spanned the entire product. Recombinants were generated per manufacturer's protocols (AdEasy, Agilent, Santa Clara, CA), selected using kanamycin resistance, confirmed by sequencing, amplified in XL10-gold ultracompetent cells, purified, PacI-linearized, and transfected into HEK293 cells to produce adenoviral vectors coding for FAK-Helix (Ad-FAK-Helix) and the FAK-HelixScr (Ad-FAK-HelixScr). Viral particles were expanded and collected per manufacturer's protocols, and passed through a Fast-Trap Adenovirus Purification and Concentration kit (EMD Millipore, Darmstadt, Germany) before reading OD at 260nm. Viral titer was calculated as one A260 unit to 1012 viral particles with a 50:1 ratio of particles to infectious particles via agarose overlay per manufacturer's protocols (not shown).

### Pressure regulation

Pressure was controlled using an airtight apparatus previously described, pressurized with filtered 5% CO2/95%, and maintaining temperature, pressure, pO2, pCO2, and pH [7].

### Cell adhesion studies

SW620 colorectal adenocarcinoma cells (ATCC, Manassas, VA) at 90% confluence were split 1:4 two days previously to achieve 50-60% confluence on the day of adhesion assay. SW620 cells were trypsinized, plated randomly at 5×10^4^ cells/well, and allowed to adhere to collagen-I-coated plates for 30 minutes at 37°C under ambient or 15mmHg increased pressure [7]. After 30 minutes, non-adherent cells were washed away with warm PBS. Ambient and pressure-treated plates were encoded to prevent treatment identification during washing. Adherent cells were incubated for 1 hour at 37°C with CellTiter 96 Aqueous One Solution Reagent (Promega, Madison, WI), and absorbance measured at 490nm with an Epoch plate reader (Biotek, Winooski, VT). For adenoviral experiments, SW620 cells were grown to 90% confluence in a T25 flask (Corning, Corning, NY) before viral infection (13x 10^3^ vp/cell) for 1 hour before replacing infection media with growth media. After 24 hours, infected SW620s were replated in new T25 flasks at a 1:4 ratio. At 72 hours, adhesion was assayed as above. Cells were used within ten passages and authenticated by ATCC.

### Phosphorylation

SW620 cells were transfected with Ad-FAK-Helix or Ad-FAK-HelixScr for 72 hours, trypsinized, and exposed to ambient or 15mmHg increased pressure for 30 minutes at 37°C in growth media in 48 well plates pacificated with 1% heat-inactivated BSA in PBS (to prevent adhesion). SW620 cells allowed to adhere to collagen-I-coated plates for 30 minutes at 37°C were positive controls. Cells were lysed for western analysis.

### Cell proliferation

SW620 colorectal adenocarcinoma cells were seeded at 10^4^ cells/well on 96 well plates, recovered at 37°C for 12 hours, allowed 24 hours for proliferation, and counted using CellTiter 96 Aqueous One Solution Reagent as above. In adenoviral studies, SW620 cells were transfected with Ad-FAK-Helix or Ad-FAK-HelixScr 60 hours before plating on 96 well plates. For nonsurvival wound adhesion studies, cells were dyed with 10μM Tag-it Violet proliferation and cell tracking dye (BioLegend, San Diego, Ca), or equivalent amounts of DMSO vehicle.

### FAK-Akt1 coimmunoprecipitation

Coimmunoprecipitations were performed as previously [10] using mouse monoclonal antibodies to Akt1 (CST, Beverly, MA) and HA (Convance, Berkley, CA).

### Transfections

SW620 cells were transfected using Lipofectamine 2000 reagent (ThermoFisher, Waltham, MA) and HA-FAK(WT) plasmid (from Dr. JL Guan). Cells were grown in T75 flasks until 80% confluent, replated into 6 well plates at 80% confluence, and transfected 12 hours later per manufacturer's protocols.

### Western blotting

Protein concentrations were determined by bicinchoninic acid (BCA) protein assay (Pierce, Rockford, IL). Eluate from GST pull-downs or collected cell lysates was resolved by SDS-PAGE and transferred to Hybond P 0.45 PVDF blotting membrane (Amersham Life Science, Arlington Heights, IL). Membranes were blocked for 1 hour at room temperature with Odyssey TBS Blocking Buffer (Amersham Life Science, Arlington Heights, IL) and blotted overnight at 4°C with antibodies against FAK (#3285), Phospho-FAK Tyr397 (#3283), Akt1 (#2967), Phospho-Akt1 Ser473 (#9271), GSK-3β (#9315), Phospho-GSK-3β Ser9 (#9315), or the GST tag (#2624) (CST, Beverly, MA). Membranes were visualized by the infrared fluorescent IRDye system (LI-COR Biosciences, Lincoln, NE) and analyzed on an Odyssey scanner (LI-COR Biosciences, Lincoln, NE) within the linear range. The doublet produced by the Akt1 antibody is consistent with results with this [50, 51] and other [52, 53] anti-Akt antibodies. Results were normalized to the appropriate GST tag, GAPDH, or total protein (for phosphorylated proteins) and relative to associated ambient controls unless stated otherwise.

Akt1 frequently appears in published Western blots (34-37) as a doublet. The reason for this is not entirely clear, but may represent altered phosphorylation states or other post-translational modifications of the molecule yet to be clarified. The cell line studied and its phenotype in the experiment in question, the amount of protein loaded and concentration of primary and secondary antibody, the percentage of the SDS-PAGE used to resolve the proteins, and numerous other factors may influence this. The densitometric data presented here considers the heavier Akt1 band in each of the doublets.

### Wound implantation

SW620 colorectal adenocarcinoma cells were transfected with Ad-FAK-Helix or Ad-FAK-HelixScr. After 72 hours, cells for nonsurvival studies were trypsinized and dyed with 10μM Tag-it Violet (BioLegend, San Diego, Ca) per manufacturer's protocols; survival studies used undyed cells. Cells were incubated for 30 minutes at 37°C under ambient or 15mmHg increased pressure in a 48 well plate pacificated with 1% heat inactivated BSA in PBS to prevent adhesion to the plate. These cells were then collected and washed in warm PBS. In nonsurvival studies, 1 cm groin incisions were made bilaterally in 6-7 week old 22.9-24.2 gram male BALB/cAnNHsd mice (Envigo, Haslett, MI) anesthetized i.p. with ketamine (100mg/kg), xylazine (10mg/kg), and acepromazine (3mg/kg). In survival studies, a single 1 cm groin incision was made in 6-7 week old 22.9-24.2 gram male athymic nude- Foxn1nu mice (Envigo, Haslett, MI) anesthetized with continuous inspired 1-2% IsoFlo (Abbott Laboratories, North Chicago, IL) in oxygen. A 50 μl suspension ambient or increased pressure of 5×10^5^ cells was randomly applied to the wounds. After 30 minutes, the fluid was aspirated and the wounds were washed with warm phosphate buffered saline six times to remove nonadherent cells. In nonsurvival studies, the mice were euthanized following wound irrigation, and wounds were excised to quantify tumor adhesion by fluorescence-activated cell sorting (FACS). The excised wound tissue was mechanically (paired scissors) and then enzymatically (3ml/sample collagenase incubation for 1hour at 37°C with agitation) disaggregated before passage through a cell strainer and 20 minute room temperature incubation in BioLegend fixation buffer (BioLegend, San Diego, Ca). Fixed cells were resuspended in PBS with 5%FBS and Tag-IT dye. Fluorescence and cellular auto-fluorescence were detected using a LSR flow cytometer (BD Biosciences, San Jose, CA) with a filter for Pacific Blue (ex/em 410/455). In survival studies, the wounds were instead closed and followed as described [9]. Animal studies were sized to yield 95% confidence with 80% power and approved by the Institutional Animal Care and Use Committee of the University of North Dakota.

### Statistics

Data are expressed as mean±SEM. Results were compared by Student's unpaired t-test and log-rank test as appropriate seeking 95% confidence. In vivo studies were analyzed by Mantel-Haenszel testing. Assays were within linear ranges.
